# Genetics of asthma: a molecular biologist perspective

**DOI:** 10.1186/1476-7961-7-7

**Published:** 2009-05-06

**Authors:** Amrendra Kumar, Balaram Ghosh

**Affiliations:** 1 Molecular Immunogenetics Laboratory, Institute of Genomics and Integrative Biology Mall Road, Delhi-110007, India

## Abstract

Asthma belongs to the category of classical allergic diseases which generally arise due to IgE mediated hypersensitivity to environmental triggers. Since its prevalence is very high in developed or urbanized societies it is also referred to as "disease of civilizations". Due to its increased prevalence among related individuals, it was understood quite long back that it is a genetic disorder. Well designed epidemiological studies reinforced these views. The advent of modern biological technology saw further refinements in our understanding of genetics of asthma and led to the realization that asthma is not a disorder with simple Mendelian mode of inheritance but a multifactorial disorder of the airways brought about by complex interaction between genetic and environmental factors. Current asthma research has witnessed evidences that are compelling researchers to redefine asthma altogether. Although no consensus exists among workers regarding its definition, it seems obvious that several pathologies, all affecting the airways, have been clubbed into one common category called asthma. Needless to say, genetic studies have led from the front in bringing about these transformations. Genomics, molecular biology, immunology and other interrelated disciplines have unearthed data that has changed the way we think about asthma now. In this review, we center our discussions on genetic basis of asthma; the molecular mechanisms involved in its pathogenesis. Taking cue from the existing data we would briefly ponder over the future directions that should improve our understanding of asthma pathogenesis.

## Introduction

The realization that asthma is a genetic disorder, which runs in families, is relatively old and can roughly be dated back to the early 20^th ^century, where investigators sought to identify traits with simple Mendelian mode of inheritance responsible for asthma pathogenesis [1]. Later, epidemiological surveys were conducted that demonstrated the heritability of asthma using twin studies [2]. Owing to the variable phenotypes that asthma presents with [3], defining it clinically has been challenging and no definitions so far have been fool proof, in terms of sensitivity and specificity [4]. The definitions and guidelines have seen transformations from time to time depending upon our understanding of its etiopathology [5]. Put in its simplest form, asthma is a chronic pulmonary disorder which is characterized by airway inflammation and remodeling that leads to reversible airway obstruction [3]. Inflammation is seen mainly in the larger conducting airways; however in severe forms of asthma even the smaller airways show infiltration of immune cells [6]. Asthma represents a spectrum of disease and apart from symptoms like wheezing and breathing difficulty; cough, running nose and eyes, dyspnoea etc. may accompany it with variable degree and frequency. Inherent genetic factors interact in complex fashion, with environmental triggers, to bring about its pathogenesis and depending upon the trigger, it is classified as extrinsic or intrinsic asthma [7]. Extrinsic asthma results from hypersensitivity reactions (such as wheal and flare reaction to intradermal allergens), resulting in increased serum IgE and bronchial hyper-responsiveness to specific or non-specific inhaled allergens [7]. In contrast intrinsic asthma is thought to be non-immune and without any atopic background. We mainly focus on extrinsic asthma, where there is plenty of genetic data to build up a sketch of the molecular biology pathways that play significant role in its pathogenesis. Also, the molecular mechanisms that these studies have unearthed have promising therapeutic potentials.

### Asthma pathogenesis: a disease of dysregulated immune system

Asthma pathology has been traditionally supposed to be an overenthusiastic response of the immune system to otherwise innocuous environmental allergens or challenges. However recent evidences suggest that most of the allergens that were thought to be innocuous have protease activity [8,9] or other deleterious effects [10] and our bodies' immune response against them might reflect an ongoing evolution of human beings/other animals with their environment. In asthma, there is infiltration of mast cells, basophils, eosinophils, lymphocytes, macrophages etc. into the bronchial mucosa and these cells along with the cells of the respiratory tracts such as epithelial cells, endothelium, smooth muscles etc. bring about airway inflammation and airway remodeling [3]. Both these components have hereditary factor and are influenced by the environment [3].

Since asthma is growing rapidly worldwide in the late 1980s, the "hygiene hypothesis" was proposed to explain the possible causes of asthma (and other related disorders) based on its increased prevalence in industrialized societies [11]. It states that lack of microbial fauna during the early developmental stages leads to immune hyperreactivity disorders [11]. Later on it was identified that T helper cell bias towards a Th2 phenotype might be responsible for asthma pathogenesis [12]. Consequently, some evidences led scientists to propose that during birth the immune system is polarized towards Th2 response while exposure to microorganism during early developmental stages drives the immune response towards Th1 type which is protective against atopic asthma [13,14]. This was established as immunological basis for hygiene hypothesis [15]. Since then Th1/Th2 polarization has become the hallmark to explain the causes of asthma [16] and not surprisingly it has dominated the field of asthma genetic research for the last two decades. Also, most of the efforts to discover novel therapeutics to treat or cure asthma have been centered on this principle [17]. Some pioneering genetic discoveries in the last few years have shifted our attention partly to other possible causative mechanisms and there is growing realization that the local tissue environment actually plays significant, if not major role in initiating asthma [18] and plays important roles in its progression and severity [19].

### Approaches to identify genetic components in asthma

Before embarking on to the genetic evidences which have provided clues regarding the molecular pathways in asthma, we should take a brief look into the methods used to fish out susceptibility genes for asthma or complex disorders. We shall not go deep into each of these methods as excellent reviews already exist for the readers to acquaint themselves with the latest techniques; biochemical, molecular, analytical [20-23] etc..

Population genetic studies like association studies and linkage studies have played major roles in identification of several causative genes for most of the complex disorders including asthma [20,23-25]. Essentially population genetic studies could be either hypothesis driven, which is the case in candidate gene studies, or with no prior hypothesis such as linkage studies. In candidate gene studies, genes are selected from the pathways shown or expected to play role in asthma pathogenesis. Candidate gene studies could be based on allele frequency differences between affected (cases) and non-affected (control) individuals known as case-control studies or based on transmission distortion or disequilibrium of allele(s) as in family based association studies [25]. Candidate gene studies are supposed to have high sensitivity to detect alleles or variants playing minor role in disease pathogenesis [21]. On the contrary, linkage studies are usually carried out with motivation to identify novel disease loci/genes by genotyping evenly spaced markers in the entire genome, in large extended families [20]. Since large fractions of genome are shared among individuals in a family, it is expected that loci with large effects on the phenotypes could be detected easily and fine mapped to fish out the susceptibility genes [20,23]. As obvious, sensitivity and specificity are two vital issues when adopting any of the two approaches. While the debate continues, development of high-throughput array based technologies, with densely mapped markers, have opened up newer avenues to perform genome wide association studies that perhaps should take care of sensitivity and specificity issues in a better way [22,23].

Other very popular approaches for disease gene identification have been microarrays, which take advantage of the fact that transcripts of various genes can be assayed at large scale simultaneously [26]. Using both human subjects and animal models a number of studies have been undertaken that have identified novel genes/pathways or validated others that play important role in asthma pathogenesis and may have therapeutic potentials [26]. Combined with animal models this technology has played pivotal role in identification of genes/molecules involved in complex diseases [25]. Animal models are suitable as confounding environmental factors can be better controlled and tissue samples can be harvested sufficiently with ease. Also, identical genetic background of the inbred animal strains allow for dissection of environmental factors in influencing gene regulation in different pathological conditions.

It should be appropriate to mention here that a plethora of genetic association or linkage studies fail to replicate in different populations, and that tend to frustrate geneticist as faith in such data has been shrinking. Arguably, as though, methodological issues pose daunting challenges, the reason for such variable discoveries could not be assigned single handedly to poor study designs, as some very well designed studies have also shown variable results [27]. In addition, ethnic variation may also account for such non-replicative results across different populations. Consequently, hunt for newer technology, newer analytical tools are on, which should address these problems in the near future [23,27]. It is unlikely that any single factor, genetic or environmental, could account for asthma pathogenesis, therefore statistical tools are being designed to carryout multifactorial analysis [27]. Also, lots of efforts are being put to develop cheaper and affordable sequencing technologies so that sequencing of large number of individuals can be carried out faster and more accurately [28]. When sequencing technologies become cheaper they would facilitate geneticists to include more individuals to give power and confidence to their observations and discoveries. Similar revolution in other related fields like proteomics, lipidomics, epigenomics etc. should accelerate the identification of genetic components and dissection of molecules and pathways relevant to asthma.

Having set the stage to start our discussion on genes, molecules and pathways it would be helpful to divide the available genetic data into two categories; genes that affect inflammation and genes that play critical role in airway remodeling events. To caution the readers, it should be mentioned here that most if not all of the genes could have multiple roles and take part in both the events. In fact it has been difficult to study these events in isolation for all practical reasons as they are tightly connected processes. However it is desirable for the purpose of making the discussion simple and interesting. Also, since excellent reviews are available in this area [20,23-27,29], we would like to highlight some recent discoveries that have been discussed less but have great potential for our understanding of asthma pathogenesis and consequently offer opportunities to design intervention strategies.

### Genes influencing the inflammatory pathways

It was around late 1980's and early 1990s, when human chromosomal regions were first found to be linked with allergy or asthma [30-32]. Since then various mediators of inflammation have been identified using approaches mentioned above [20,23-27,29]. Several genome-wide screens have found linkage to chromosomal regions, such as, *5q23-31, 5p15, 6p21.3-23, 11p13, 11p15, 12q14-24.2, 13q21.3, 14q11.2-13, 17p11.1-q11.2, 19q13, 21q21 *etc. [20,23-27,29,33]. The most consistently replicated among them are *5q23-31, 5p15 *and *12q14-24.2 *containing genes like *IL-3, IL-4, IL-5, IL-9, IL-12b, IL-13, IFNγ, iNOS, FCεRIβ *etc. [23,33]. Most of these influence the T cell development/polarization towards Th1 or Th2 besides modulating other features like recruitment of eosinophils, mast cells, neutrophils etc. to the site of inflammation [23,24,33]. These genes have also been validated using candidate gene approaches in different studies and a number of functional polymorphisms have been identified. It was found that the polymorphisms in the intronic region of *IFNγ *gene may be critical for *IFNγ *gene regulation and atopic asthma [34]. Similarly inducible nitric oxide synthase or iNOS which is expressed predominantly by immune cells and epithelial cells harbor a number of promoter and intronic polymorphic repeats that could be regulating its expression and asthma related traits [35]. Importantly, we had identified an intron 4 repeat to be associated with asthma severity [35].

Candidate gene approaches have also led to identification of some important genes that play critical role in asthma pathogenesis. For example, AMCase or acidic mammalian chitinase is present on outer coating of several organisms like fungi arthropods etc. and is found associated with asthma by our lab [36] and others [37]. Polymorphisms in *FCε RIβ *show association across different population [23]. In Indian population, we had identified protective and risk haplotypes that regulate IgE mediated histamine release [38,39]. Several other genes playing role in innate immune recognition and immunoregulation, antigen presentation, biosynthesis and regulation of lipid mediators, IgE synthesis and regulation, Th2 differentiation and effector function, and other pathological mechanisms have been identified and discussed elsewhere [20,23-27,29,33].

As mentioned earlier T helper cell differentiation play vital role in asthma pathogenesis. Recently another T helper subset, namely Th17, has been discovered [40] and the mechanism of its development, differentiation etc. has been studied in good detail [41]. While initially discovered to be mediating autoimmune disorders [40], some recent finding suggest that it might be playing very significant role in inflammatory pathways critical of asthma pathogenesis [6,42,43]. IL-17 is the effector cytokine produced by Th17 cells, and has increased concentration in asthmatic sputum [42]. Recently, Kawaguchi et al have reported one coding-region sequence variant, His161Arg substitution in *IL-17 *gene, which is associated with protection against asthma [44]. They also demonstrated using in-vitro studies that this polymorphism inactivates the ability of this cytokine to activate mitogen-activated protein kinase, thereby acting as natural antagonist [44]. Th17 cell also secret IL-21 which helps in its differentiation and mediates its effector functions [40]. IL-21 has been shown to regulate IgE synthesis and it has been shown that one exonic variant *C5250T *in exon 3 of this gene is associated with asthma and serum total IgE [45]. This polymorphism might be affecting mRNA structure as our bioinformatics results suggest [45]. The role of Th17 in asthma pathogenesis, however, needs further investigations, as extrapolations from inflammatory event involved in autoimmune diseases suggest that it could be playing vital role in its pathogenesis, since it suppresses the development of regulatory T cells and their action [6].

PI3K plays critical role in the inflammatory events and shown to modulate multiple features of asthma such as mast cell development, migration and degranulation, eosinophil migration and activation, T cell differentiation, B cell activation, IgE synthesis and production etc. [46,47]. In immune cells PI3K mediates its action through phosphoinositol 3, 4, 5 tri-phosphate, which acts as messenger and recruits various downstream molecules constituting a signallosome [47]. Several phosphatases have been identified that dephosphorylate this lipid messanger and downregulates PI3K signaling in immune cells [47]. SHIP (src homology 2-containing inositol phosphatase) is 5' phosphatase and it downregulates mast cell degranution upon IgE crosslinking, therefore it could regulate asthma pathogenesis [48]. PTEN (phosphatase and tensin homologue) which is 3' phosphatase has been shown to downregulate IL-4, IL-5 and eosinophilic cationic protein that are expressed in ovalbumin challenged mice [49]. Also, PTEN reduces vascular endothelial growth factor (VEGF) expression in allergen induced airway inflammation [50]. Taking lead from differentially expressed genes in a microarray study in ovalbumin sensitized mice, we have recently identified inositol polyphosphate 4-phosphatase type I (*INPP4a*), a novel gene associated with asthma, using population genetics as well as in-vitro and in-vivo studies [51]. This study lead to the identification of a non-synonymous SNP *+110832 A/G *(Thr/Ala) within a PEST (proline, glutamic acid, serine and threonine) enriched region to be significantly associated with asthma. Further, on western blot analysis using human platelets isolated from human peripheral blood, it was demonstrated that this polymorphism affects INPP4a stability, as threonine to alanine substitution, possibly resulted in less degradation of INPP4a by calpain mediated proteolysis [51]. INPP4a dephosphorylates and inactivates phosphoinositol 3, 4, bis-phosphate preferentially, another important messanger in the PI3k-akt pathway. Therefore, SHIP, PTEN and INPP4a seem to be major players in regulating PtdIns(3,4,5)P_3 _degradation pathway and, in our view, hold promising therapeutic potential. It is very appealing to propose here that these molecules should be intricately regulated and must be interacting to keep harmful effects of PI3K at bay; it would be interesting to empirically demonstrate this. It is also very logical to propose that INPP4a, being the terminal enzyme, could play a major role [51].

### Genes involved in airway remodeling

Unlike inflammation in asthma, airway remodeling component has not received much attention as earlier it was believed that it appears late in disease process, resulting from persistent inflammation. However, there are reports which suggest that airway remodeling events are evident even prior to the development of disease process in individuals with asymptomatic AHR [52]. Airway remodeling refers to the structural changes of the surface of the airway that lead to its narrowing and constriction. Earlier studies demonstrated that it might have a role to play in severe asthma but recent studies suggest that some aspects of it are present in all forms of asthma at every stage of disease progression [53,54]. Identification of ADAM33, a disintegrin matrix metalloproteinase 33, was the beginning that lead researchers to believe that airway remodeling events are quite distinct and are influenced by genetic factors. *ADAM 33*, which is present on chromosome 20, was identified by positional cloning approach, using linkage studies in a Caucasian population [55]. Several studies have replicated this in different populations asserting its importance [23]. It is expressed by lung fibroblasts and bronchial smooth muscles but not by bronchial epithelial or immune cells [56]. ADAM proteins have many domains and, they have several forms that play various roles in immune system [56]. The functional role of ADAM33 is speculative at present and to be demonstrated experimentally [57]. *DPP10 *(dipeptidyl peptidase 10) is another gene that was identified, using positional cloning approach in mouse and human, to be associated with brochial hyperresponsiveness and IgE [58]. This gene is located on chromosome *2p14 *and encodes for a member of dipeptidyl peptidase family of proteins and acts to limit the activity of proteins like cytokines, leukotrienes etc. which have key roles in asthma pathogenesis [59]. *GPRA *(G protein coupled receptor for asthma), which is located on chromosome *7p15 *also shows consistent association with asthma after its initial linkage to asthma related traits [60]. GPRA isoforms are differentially expressed in brochial epithelium and airway smooth muscle of asthmatics and normal controls [60]. *SPINK5*, on chromosome *5q23-31*, is another gene that might play an important role in airway remodeling as it is highly expressed in bronchial epithelium and consistently shows association with asthma [18]. The role of TGFβ in airway remodeling is well documented in genetic [61] and immunological studies [62]. In Indian population, we had identified specific haplotypes to be associated with asthma and serum TGF levels, indicating that polymorphisms play important role in regulating TGF levels [61]. Taken together, these data suggest a vital role of tissue remodeling, in asthma pathogenesis, which is brought about by complex interaction of tissue components like epithelium, smooth muscles etc. These evidences indicate that therapeutic interventions must be sought for airway remodeling events which might not be taken care of by the present therapeutic regimen. Increased prevalence of severe asthmatics may be explained by the hypothesis that lack of treatment of airway remodeling during early stages might make the disease more severe in the later stages. Not surprisingly, some of the genes stated above also show association with severe form of asthma, particularly *ADAM33 *[63].

Involvement of mitochondria in asthma pathogenesis is under investigation and receiving considerable attention. In animal models and human children, increase in mitochondrial number and altered mitochondria has been reported [64,65]. The increase in mitochondrial number or mitochondrial biogenesis is calcium dependent, regulated by a number of mitochondrial factors [66]. Further, we have recently demonstrated that mitochondrial structural changes leading to its dysfunciton plays a critical role in asthma pathogenesis [67]. Also, mitochondrial dysfunction is IL-4 dependent, since mitochondrial structure and associated changes, could be reversed by IL-4 mAb [67]. Additionally, it has been shown that mitochondrial factors play crucial role in modulating neutrophil survival in atopic asthmatics [68]. Since there are reports of maternally inherited asthma [69] and mitochondria is believed to be inherited only from the mother, mitochondrial genes could be playing an important role in asthma pathogenesis. From mite induced and uninduced peripheral blood mononuclear cells of mite sensitive allergic patients, Tochigi-ken et al identified 13 differentially expressed genes using substractive hybridization, 9 of which were mitochondrial genes [70]. Also, Raby et al have demonstrated association of a mitochondrial haplogroup with serum IgE in 654 white children with mild or moderate asthma [71]. More well designed studies in future, in different populations, should provide further evidences on the role of mitochondrial gene polymorphisms in contribution to genetics of asthma, since mitochondria is critical player in modulating apoptosis [72].

Well, we can definitely be convinced that we know a great deal about asthma relative to what we knew few years back, but still, as we have come across throughout the text, problems loom larger. Asthma seems to be increasing not only in frequency but also in the intensity or severity of its affection and that is worldwide phenomena [63]. Severe or refractory asthma, as it is known, constitutes nearly 10% of asthmatics, which are sizable proportions that remain uncontrolled or poorly controlled [63]. In fact knowing more about it, only one thing becomes clear and that is, we probably are too far from its comprehensive understanding. Even now, the most preferred therapy remains the use of steroids and β_2_-agonist which had been discovered long time back and, although, their efficacy has been improved in the past few years, these are mere symptomatic cure and do not help in managing all kinds of asthma, besides their reported side effects [73,74]. However it should be mentioned that some excellent approaches have been attempted at, like allergen specific immunotherapy or immunotherapy using CpG oligonucleotides to help strengthen the immune system. Also, some of the candidates like TNF, IFNγ, IL-4, kinases etc. have been made targets and agonists and/or antagonist developed/discovered to ameliorate asthma [73,74]. These cytokines/chemokines/kinases etc. have pleiotropic and/or redundant functions and trying to play with them seem to be very detrimental to the normal immune homeostasis [73,74]. Omalizumab, the humanized anti-IgE had shown promising results in the clinical trials but it is very costly and unlike steroids they are not effective against large sections of asthmatics [75].

These problems, are certainly, not limited to asthma but other complex disorders as well. The opportunities lie in trying to capture the complex interaction between molecules and pathways that cause asthma. Very interesting and logical suggestions have been forthcoming in this direction and we will discuss some of them briefly.

### Gene-gene and gene-environmental interactions; towards multifactorial approaches

Asthma is a multigenic disorder and is greatly influenced by environmental factors, as we have seen in our earlier discussions. Therefore testing for a single gene or single factor for accurate prediction of disease outcome is an unjustified expectation [76-78]. In fact, analyzing for a single locus for traits that are controlled by multiple loci, there is considerable loss of power, depending upon the underlying genetic model used [79]. In a linkage study involving three ethnic groups from USA, Jianfeng Xu et al report significant increase in LOD score for several loci in their gene-gene interaction analyses [80]. For example, evidence of linkage at 5q31 increase from LOD score 0.98 to 3.21 when analysis was conditioned upon linkage at 1p32 [80]. Other loci such as 12q22, 8p23, 15q13 also showed increased LOD score when their analyses were conditioned upon loci that had showed marginal signals in their independent analyses. These results were also complemented by affected sib-pair two loci analysis [80]. Several other studies in asthma and other complex disorders suggest that gene-gene interaction studies could enhance disease outcome prediction when, concurrently, genes from a pathway or interacting pathway are selected [81,82]. Similarly, different environmental factors (physical, chemical, nutritional, behavioral etc.) have been studied in isolation and shown to affect asthma and related phenotypes but their interaction effects have been missed [83]. Environmental factors act like rheostat and influence gene regulation/expression. We do not inherit disease state per se but a set of susceptibility genetic factors often respond to environmental stimuli and predispose individuals to a higher risk group. Our studies, therefore, should also take into account gene-environment interactions and its influence on complex diseases like asthma. Polymorphisms in 17q21 confer higher risk in early onset asthma and the risk increases further when there is exposure to environmental tobacco smoke in early life [84]. This region contains four genes all of which could have potential role in asthma pathogenesis [84]. Guerrera S. et al rightly point out that we have to take a paradigm shift and design studies that take into account multiple factors that could be partners in bringing about disease pathogenesis [27]. Also well planned phenotyping strategies would greatly enhance outcome prediction in complex heterogeneous disease like asthma [27]. However, current analytical tools have limitations with regard to number of parameters (genetic/environmental etc) that could be included in interaction analysis since increase in parameters result in increase in dimensionality of the data. Traditionally, logistic regression analyses have been performed to identify interacting partners but they do have limitations since only parameters having independent primary effect could be tested for interactions. Approaches such as multifactor dimensionality reduction etc are non-parametric tests that could identify interaction even in the absence of independent primary effects and are becoming very popular for performing gene-gene and gene-environment interactions. It is expected that in future low cost genotyping along with statistical tools that handle high dimensional data would revolutionize this field.

### Epigenetics

Epigenetics, the term, that refers to heritable characters other than those encoded in the DNA sequence, play major role in gene-expression [85]. Epigenetic silencing, which is mediated by DNA methylation, histone modifications and small RNAs, is influenced by both genetic and environmental factors [85]. These epigenetic changes could also be inherited transgenrationally influencing disease susceptibility [86]. Epigenetic studies have potential to demonstrate the gene expression changes that occur during disease processes, for example the epigenetic changes accompanying T helper cell differentiation towards Th1 or Th2 have been described [87]. Methylation changes in the promoter and intronic regions of *IL-4 *gene have been shown to modulate the production of IL-4 [88]. Similarly hypermethylation in the *IFNγ *gene leads to higher production of IL-4 due to suppressed production of IFNγ [89]. These two genes are critical modulators of Th1/Th2 balance and play vital roles in asthma pathogenesis. Also, it has been shown that untreated subjects with asthma possess higher levels of histone acetyltransferase (HAT) and lower levels of histone deacetylase (HDAC) in bronchial biopsies which get reversed upon steroid administration [90]. Similar observations have also been made for COPD which has many feature common to asthma. [91]. Parent of origin effect has also been noted wherein polymorphisms inherited from a particular parents (father or mother) influence the disease susceptibility of the offspring [86]. In this regard maternal prenatal environment seems to play vital role in bringing about gene expression changes in the offspring [86]. Many epidemiological studies point towards critical role that prenatal and early postnatal environmental exposures could play in bringing about asthma pathogenesis [86]. For asthma which has variable time of onset it has been proposed that certain epigenetic changes during adulthood could also influence the disease onset and progression [86]. Micro RNAs (miRNAs) have emerged as critical players of gene regulation, post-transcriptionally and post-translationally and could be key mediators of epigenetic regulation [92]. It has been shown that a single nucleotide polymorphism in HLA-G gene affects binding of three different miRNAs to this gene [93,94]. Recentally from our lab it has been demonstrated that miR-106a brings about post-transcriptional regulation of IL-10 gene expression. Expression of miR-106a is modulated by transcription factors egr1 and sp1 which binds to miR-106a promoter [95]. *IL-10 *is an important candidate gene found to be associated with asthma in many population genetic studies [96]. It is worthwhile to note that a number of miRNAs have been shown to have critical role in immunity [97]. Till now nearly 300 miRNAs have been identified and each of them could target hundreds of genes [98]. Recent development of technologies that enable high-throughput/genome-wide detection of epigenetic changes should bring out more data relevant to asthma and related phenotypes. It should be vital to know how genetics, environmental factors and epigenetics regulate each other and in turn the molecular events that underlie complex diseases such as asthma.

### Copy number variation/polymorphisms

The genomic variation in the human genome ranges from single nucleotide variation to large microscopically detectable variations that have also been shown to be associated with many disorders [99]. The advancement in the genotyping technology have led to identification of structural variation that fall in between these two extremes, known as copy number variations (CNVs) [99]. Currently all genomic variations larger than 1 kb of DNA are termed as structural variations. Structural variants could lead to change in gene dosage in case of deletion or duplication etc. or with any change in gene dosage as in inversions or balanced translocation [99]. Initially identified in case of sporadic disorders, inherited CNVs have been reported and associated with many infectious and immunological disorders like, HIV, systemic lupus erythomatosus, lupus glomerulonephritis etc. [99]. Various issues related to identification and analysis of copy number polymorphisms are being debated and under modification [100]. However, it has generated enthusiasm among geneticists as it has potential to explain gene dosage changes in some of the complex disorders [101-103]. Asthma like other complex disorders should certainly benefit from this field and more and more genetic components could be identified.

## Concluding Remarks

In the last few decades the efforts to understand the pathophysiology of asthma has been intensified due to its increasing morbidity and mortality. The need to understand the genetics of complex disorders has led to much advancement in the technologies that have contributed to our increased understanding of asthma as well. However, we still have a long way to go, before the available data is assimilated to design effective intervention strategies and check asthma menace. We have attempted here to summarize the contribution of genes in asthma and what pathways these genes belong to. We need to put more focused efforts to chalk out molecular pathways and draw a comprehensive map of molecular interactions that underlie asthma pathogenesis. In this regard, we have outlined strategies that should be filling up the missing link together with possibilities of revolutionary findings. Although, not very sure where we stand, we definitely are inching closer and we should identify some novel therapeutic strategies that could lead to better asthma management and perhaps cure.

## Competing interests

The authors declare that they have no competing interests.

## Authors' contributions

AK, senior research fellow works on identification and validation of targets for asthma. BG is the head of division of Molecular Immunogenetics, IGIB-CSIR, India. He has been working on genetic and molecular biology of asthma pathogenesis and mentor of AK.
